# Economic issues of Severe Acute Respiratory Infections for influenza in Mexican children attended in a tertiary public hospital

**DOI:** 10.1371/journal.pone.0273923

**Published:** 2022-09-09

**Authors:** Alfonso Reyes-Lopez, Sarbelio Moreno-Espinosa, Yosef Olaf Hernandez- Olivares, Jimenez-Juarez Rodolfo Norberto

**Affiliations:** 1 Center for Economic and Social Studies in Health, Federico Gómez Children’s Hospital of Mexico, Mexico City, Mexico; 2 Teaching Directorate, Federico Gómez Children’s Hospital of Mexico, Mexico City, Mexico; 3 Department of Infectious Diseases, Federico Gómez Children’s Hospital of Mexico, Mexico City, Mexico; 4 Department of Paediatrics, Hospital of Infectious Diseases, La Raza National Medical Center, Mexican Institute of Social Security, Mexico City, Mexico; University of South Florida, UNITED STATES

## Abstract

**Background:**

Influenza cause a clinical and economic burden for health systems and society. It is necessary to know the cost of the disease in order to perform cost-effectiveness assessments of preventive or treatment interventions.

**Objective:**

Assess the costs of the care of children with influenza in a third level hospital in Mexico.

**Methods:**

Longitudinal retrospective study based on the review of clinical files of children hospitalized with influenza. The use of resources used during their hospitalization in the emergency room, general ward, or PICU was logged, and the amount of supplies were multiplied by their corresponding prices to calculate the direct medical expenses. Descriptive statistics were used, and a GLM was adjusted in order to assess the effect of the clinical characteristics of the patients on the cost. Goodness of fit tests were performed.

**Results:**

132 files were reviewed, out of which 95% were of subjects who had comorbidities. Subjects admitted at the PICU generates the highest cost (mean $29,608.62 USD), when analyzing the total cost summarizing the three clinical areas (Emergency room, general ward and PICU) by age group, the highest cost was for patients over age 10 (mean $49,674.53 USD). Comorbidities increase the cost of hospitalization by $10,000.00 USD.

**Conclusions:**

Influenza causes a significant financial burden on the health system. Children with comorbidities increase the costs and children over 10 years uses a significant amount of resources and they are not a priority in immunization program. It is necessary to perform studies on the use of resources in the first and second attention levels, which represent the highest incidence of the disease.

## Introduction

Influenza is one of the respiratory viruses that causes an important disease burden throughout the world [1], and it is also the only epidemic respiratory virus that can be prevented with a vaccine. The WHO acknowledges that the decisions related to the prevention strategies must be assessed locally in accordance with each country’s income; therefore, the economic assessment for infectious diseases and their interventions (prevention or treatment) are vital for ensuring the access for the most vulnerable populations., for this proposal the WHO has even created guidelines for the economic assessment of influenza [2].

The distribution of the cases has a pyramid shape, with the mildest cases at the bottom and the most severe cases at the top, including deaths [1]. The WHO Guidelines for calculating the burden of the disease related to seasonal influenza suggests performing these calculations in cases that have been confirmed by the laboratory. For this purpose, high income countries have developed epidemiological surveillance networks and keep electronic files that allow them to assess the use of the resources for economic assessments^2^, but this is not the case in low and middle-income countries [2]. According to the local epidemiological data the annual cases of influenza in the mexican pediatric population are 2435 for children lower than one years-old, 7290 for the 1–4 age-group, 8953 for the age-group 5–9 and 21650 for children in the group 10–19 years-old [3]. Unfortunately there are no official cost estimations of influenza in this population.

Economic assessments for influenza have been performed in two arms: 1) the cost of the disease, and 2) the cost-effectiveness of the vaccination programs [4]. Economic burden information of influenza for Latin America is scarce, which in the last 15 years has resulted in a vaccination policy focused on high-risk individuals. In USA the vaccination policy changed into a universal vaccination for pediatric patients [5]. The current period of economic recession provoked by the COVID-19 pandemic has overwhelmed the health services, whereby the local governments must carry out higher investment in [4,5] vaccination in order to prevent a possible syndemic in the following years (caused by the decrease of the non-pharmacological prevention measures for COVID-19, shortages in COVID-19 vaccines along with a possible circulation of epidemic virus) causing a greater tension in health systems and a greater number preventable deaths.

The purpose of this study was to assess the healthcare economics of children with influenzain a high specialized pediatric hospital in Mexico.

## Methods

This was a retrospective longitudinal study of children hospitalized with influenza at the Hospital Infantil de México Federico Gómez. This Institute is a high specialized hospital for children which has 20 emergency room beds, 20 Pediatric Intensive Care Unit (PICU) beds, and 30 Neonatology beds, included 12 NICU beds.

Children with influenza detected through multiplex PCR, which is the standard of care for children with SARI since 2013, were identified from the database of the laboratory of molecular biology. We reviewed all the files of children who were diagnosed with influenza, and only included those that matched with the definition of SARI, excluding children with nosocomial-acquired influenza. We collected all data related to the use of the resources during hospitalization, as well as length of stay, the use of mechanical ventilation, viral co-infection and death. The information about unit cost of was extracted from the financial department of the hospital. The total cost of the care calculated by multiplying the amount of resources by their corresponding unit costs and summing these results over all categories.

All the economic results were reported in United States Dollars, using the average exchange rate during the first semester of 2020 (21.63 Mexican Pesos per Dollar).

### Definitions

Severe Acute Respiratory Infection (SARI) is defined as a recent onset acute respiratory infection, with fever (≥ 38°C), cough, and breathing difficulties that require hospitalization. Nosocomial influenza was defined as the subject who began showing compatible symptoms 48 hours after being admitted to the hospital [6].

### Econometric analysis

Descriptive statistics was performed for cost variables according to age groups, with emphasis on variability statistics to evaluate the probability distribution of costs that commonly show great variability. Cost comparisons between age groups were done using nonparametric tests. In order to assess the effect of the patient characteristics on the total cost of care, generalized linear models (GLM) methodology was used, which are models with very desirable properties not only for addressing the problem with biased data, but also for directly modelling heteroscedasticity, allowing for the possibility of having a specification that approaches the real process for generating economic data for the healthcare sector [7]. Unlike the ordinary linear regression model, GLM allow the expectation of the result variable y to be a function (known as link function) of the covariable linear index $x_{i}^{\prime }\beta$; furthermore, GLM allow for the variance of *y* to be a function of its value predicted when choosing an adequate distribution family, therefore naturally including heteroscedasticity. The link function, *g*, relates the mean costs, *y*, with the covariable linear index, $x_{i}^{\prime }\beta$, as follows:

$$x_{i}^{\prime }\beta =g\{E(y_{i}|x_{i})\}$$

The inverse of *g* maps the $x_{i}^{\prime }\beta$ index on the expected value, *μ*, conditioned over the characteristics seen for variable *y*:

$$\mu_{i}=E(y_{i}|x_{i})=g^{-1}(x_{i}^{\prime }\beta )$$

The variance, *v*, of the result variable *y* in its normal scale, is in itself a function of the mean, *μ*, but not of the covariables, except through the mean function, $\mu (x_{i}^{\prime }\beta )$. In order to select the link function and the distribution family, the performance of alternative GLM was assessed using the Akaike and Bayesian information criteria, which offers two advantages. First, they can be applied whether or not complex adjustments were performed for the purposes of data design; second, the choices based on the information criteria are not affected by the multiple hypotheses tests problems that are inherent to traditional statistics tests repeated for many possible choices. All the procedures were performed on the Stata software, version 16 [8].

### Ethics

This study was registered and approved by the Ethics and Research Committees of Hospital Infantil de México Federico Gómez under number HIM/2017/132.

## Results

**Fig 1:**
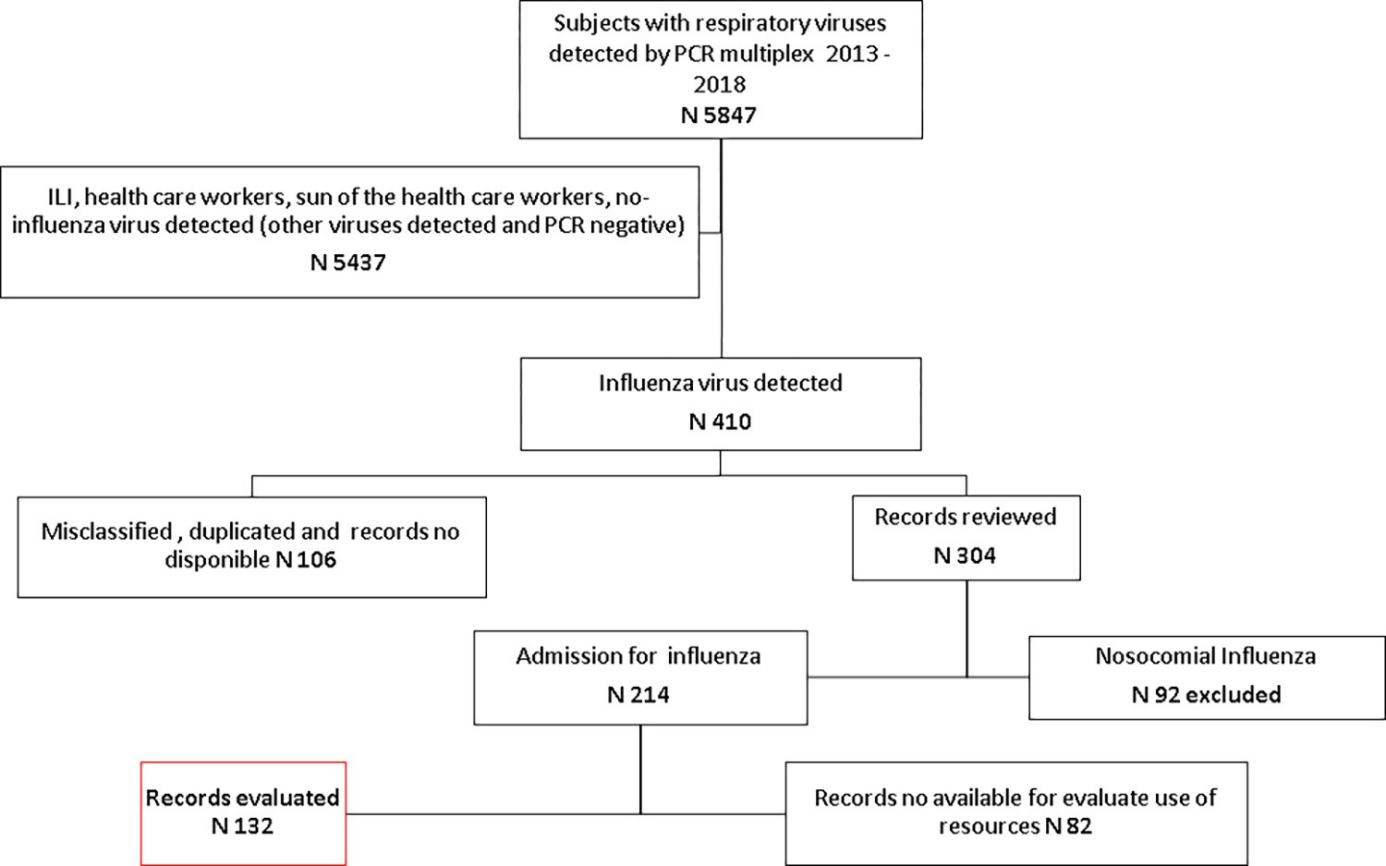
Diagram of selection of records to study.

In five influenza seasons, 5847 multiplex PCR tests were performed to detect respiratory virus. Based on these e-data, we identified and reviewed 410 files belonging to patients hospitalized with influenza. Once the duplicate, unavailable or nosocomial influenza files were eliminated, 132 files were available for the assessment of the use of resources (Fig 1).

The clinical characteristics of the patients by age group are summarized in Table 1. Out of these, 42.86% were women, median age was 5.07 (IQR 25–75 2.15–8.95), 95.45% of the children who were hospitalized had a comorbidity and only 5 (3.78%) had received the influenza vaccine for the hospitalization season (Table 1).

**Table 1:** General patient characteristics.

Characteristic	<1 year old n = 14	1 –<5 years old n = 50	5- <10 years old n = 43	≥10 years old n = 25	P value
Women, n (%)	6 (42.86)	20 (40)	18 (41.86)	13 (52)	0.79
Comorbidities, n (%)	13 (92.86)	47 (94)	41 (95.35)	25 (100)	0.64
Cardiovascular diseases, n (%)	9 (64.29)	13 (26)	1 (2.33)	Zero	0.001
Pulmonary disease	3 (21.43)	9 (18)	3 (6.98)	2 (8)	0.267
Neoplasia	Zero	11 (22)	20 (46.51)	9 (35)	0.004
Kidney disease	Zero	5 (10)	4 (9.30)	4 (16)	0.45
Congenital malformation	7 (50)	13 (26)	4 (9.30)	Zero	0.001
Perinatal	3 (21.43)	9 (18)	2 (4.65)	1 (4)	0.076
Influenza AH1N1	7 (50)	19 (38)	5 (11.63)	2 (8)	0.001
Influenza AH3N2	1 (7.14)	14 (28)	18 (41.86)	9 (36)	0.091
Influenza B	3 (21.43)	8 (16)	16 (37.21)	9 (36)	0.701
Influenza A not typed	2 (14.3)	6 (12)	3 (6.9)	3 (12)	0.813
Influenza C	1 (7.1)	0 (0)	2 (4.6)	2 (8)	0.295
Influenza vaccination history Yes, n (%) No Unknown	0 (0) 10 (71.4) 4 (28.6)	3 (6) 29 (58) 18 (36)	2 (4.6) 16 (37.2) 25 (58.1)	0 (0) 11 (44) 14 (56)	0.141
Mechanical ventilation	4 (28.57)	12 (24)	Zero	3 (12)	0.004
Admitted to the PICU	2 (14.29)	5 (10)	2 (4.65)	5 (20)	0.245

PICU: Pediatric Intensive Care Unit.

Fourteen (10.61%) children required hospitalization at the PICU. The average stay was of 9 days (95%CI 5–13); 19 (14.39%) children required mechanical ventilation with an average duration of 4 days (95%CI 2.01–5.99). The average stay at the hospital was 17.04 days (95%CI 12.10–22.71), median 6.7 (IQR 25–75 3.6–10) and the intra-hospital mortality was 1.51% (Table 1).

Tables 2–4 show different centrality, dispersion, and position statistics of the direct medical costs for these patients by age groups in order to assess the characteristics of their corresponding probability distributions. Results are presented separately depending on the hospital area where the patients were treated for (emergency room, general ward, PICU). The items that make up the total cost (duration of the stay, antibiotics, general drug product, laboratory and imagery studies, and medical procedures) are broken down for each area and age group.

**Table 2:** Direct costs for patients treated for in the emergency room (USD).

Age groups	Statistics	Bed-hours	Antimicrobials	General medication	Imaging tests	Lab tests	Procedures	Total
< 1 years old (n = 14)	Mean	$1,108.67	$85.32	$5,332.15	$6.86	$51.92	$214.11	$6,799.03
	SD	$1,068.35	$188.14	$12,111.57	$7.78	$83.13	$400.54	$12,396.43
	25th IQR	$284.24	$0.00	$0.00	$0.00	$0.00	$15.03	$867.74
	Median	$837.91	$0.21	$22.21	$5.62	$21.80	$25.87	$1,348.68
	75th IQR	$1,492.25	$57.05	$5,508.15	$11.23	$60.66	$62.32	$6,205.62
1 to <5 years old (n = 49)	Mean	$1,077.70	$438.24	$12,222.16	$11.61	$70.26	$199.94	$14,019.90
	SD	$1,261.82	$1,498.00	$33,667.90	$30.79	$90.42	$317.68	$33,909.87
	25th IQR	$284.24	$1.84	$53.82	$0.00	$9.80	$0.00	$1,264.71
	Median	$876.40	$12.78	$4,026.41	$11.23	$25.29	$30.05	$5,861.08
	75th IQR	$1,136.95	$95.38	$7,903.90	$11.23	$112.21	$346.65	$10,919.41
5 to <10 years old (n = 41)	Mean	$572.23	$346.20	$15,898.85	$10.43	$40.84	$66.89	$16,935.45
	SD	$671.11	$2,039.64	$43,604.07	$47.29	$33.71	$142.04	$43,657.79
	25th IQR	$256.60	$1.46	$14.37	$0.00	$11.00	$0.00	$868.98
	Median	$284.24	$10.19	$5,321.74	$0.00	$35.64	$15.03	$6,124.05
	75th IQR	$570.45	$45.59	$9,532.52	$11.23	$73.32	$45.08	$10,428.59
10+ years old (n = 25)	Mean	$547.51	$75.80	$18,623.96	$7.75	$97.29	$124.19	$19,476.51
	SD	$511.72	$125.76	$26,797.93	$9.20	$173.75	$264.16	$26,951.42
	25th IQR	$284.24	$2.12	$306.46	$0.00	$11.00	$0.00	$1,250.72
	Median	$285.62	$35.64	$3,952.18	$0.00	$54.92	$15.03	$4,117.83
	75th IQR	$722.30	$90.40	$28,080.77	$11.23	$73.32	$30.05	$29,148.61
Total (n = 129)	Mean	$817.66	$300.45	$13,883.63	$9.97	$64.16	$144.51	$15,220.37
	SD	$985.66	$1,473.42	$34,402.41	$32.86	$101.01	$277.93	$34,516.99
	25th IQR	$284.24	$1.46	$29.40	$0.00	$9.80	$0.00	$1,008.74
	Median	$568.48	$11.10	$3,952.18	$0.00	$33.80	$15.03	$4,776.21
	75th IQR	$1,041.22	$74.90	$10,450.01	$11.23	$78.78	$77.35	$13,924.19

SD: Standard deviation; IQR: Interquartile range.

**Table 3:** Direct costs for patients treated for in the general ward (USD).

Age groups	Statistics	Bed-days	Antimicrobials	General medication	Imaging tests	Lab tests	Procedures	Total
< 1 years old (n = 7)	Mean	$507.56	$500.46	$24,633.97	$6.42	$36.21	$153.21	$25,837.83
	SD	$180.83	$1,100.39	$64,383.34	$16.98	$40.55	$187.50	$64,301.31
	25th IQR	$355.29	$11.45	$1.46	$0.00	$0.00	$0.00	$1,069.98
	Median	$426.35	$86.78	$117.91	$0.00	$9.80	$89.51	$1,292.33
	75th IQR	$639.53	$277.43	$1,391.99	$0.00	$86.59	$323.12	$3,343.34
1 to <5 years old (n = 32)	Mean	$595.12	$1,036.93	$16,297.64	$15.71	$99.94	$85.84	$18,131.17
	SD	$569.54	$3,082.12	$45,022.08	$51.23	$262.11	$182.31	$45,356.45
	25th IQR	$355.29	$27.51	$11.32	$0.00	$3.37	$0.00	$760.09
	Median	$426.35	$78.77	$703.47	$0.00	$10.40	$40.50	$2,114.10
	75th IQR	$568.47	$238.01	$7,291.83	$0.00	$72.65	$89.51	$13,097.77
5 to <10 years old (n = 27)	Mean	$463.20	$1,744.53	$14,289.83	$0.42	$55.66	$63.42	$16,617.05
	SD	$263.21	$6,655.21	$21,618.19	$2.16	$55.97	$72.78	$22,421.31
	25th IQR	$284.23	$73.57	$5.18	$0.00	$9.80	$0.00	$600.29
	Median	$355.29	$129.07	$1,273.73	$0.00	$39.67	$89.51	$5,480.07
	75th IQR	$639.53	$380.44	$23,502.08	$0.00	$89.78	$89.51	$28,951.49
10+ years old (n = 19)	Mean	$460.01	$1,399.27	$11,144.83	$1.18	$58.59	$87.06	$13,150.95
	SD	$267.32	$4,698.56	$27,374.83	$3.54	$86.45	$137.65	$27,205.07
	25th IQR	$213.18	$25.66	$17.84	$0.00	$0.00	$0.00	$1,011.75
	Median	$497.41	$224.69	$416.61	$0.00	$22.75	$0.00	$1,845.49
	75th IQR	$639.53	$461.36	$4,318.06	$0.00	$85.07	$89.51	$6,760.99
Total (n = 85)	Mean	$515.80	$1,298.51	$15,194.58	$6.84	$71.38	$84.54	$17,171.66
	SD	$403.48	$4,709.32	$36,894.98	$32.31	$169.03	$144.97	$37,136.29
	25th IQR	$284.23	$30.05	$10.11	$0.00	$9.80	$0.00	$758.69
	Median	$426.35	$106.09	$543.09	$0.00	$19.60	$89.51	$1,930.00
	75th IQR	$568.47	$329.68	$9,051.19	$0.00	$78.96	$89.51	$17,919.83

SD: Standard deviation; IQR: Interquartile range.

**Table 4:** Direct costs for patients treated for in the intensive care unit (USD).

Age groups	Statistics	Bed-days	Antimicrobials	General medication	Imaging tests	Lab tests	Procedures	Total
< 1 years old (n = 2)	Mean	$284.23	$2,115.68	$90,758.83	$89.78	$99.68	$437.68	$93,785.89
	SD	$200.98	$2,909.21	$123,887.80	$111.08	$70.68	$110.56	$127,148.90
	25th IQR	$142.12	$58.56	$3,156.95	$11.23	$49.70	$359.50	$3,878.02
	Median	$284.23	$2,115.68	$90,758.83	$89.78	$99.68	$437.68	$93,785.89
	75th IQR	$426.35	$4,172.80	$178,360.70	$168.33	$149.65	$515.86	$183,693.80
1 to <5 years old (n = 5)	Mean	$298.45	$5,099.51	$7,933.55	$40.86	$333.58	$380.65	$14,086.60
	SD	$105.40	$4,993.86	$9,592.18	$9.91	$98.64	$539.30	$8,747.94
	25th IQR	$284.23	$2,985.91	$2,996.09	$44.94	$319.88	$0.00	$8,212.50
	Median	$284.23	$3,348.86	$4,051.34	$44.94	$332.96	$0.00	$8,677.52
	75th IQR	$355.29	$5,658.81	$7,688.49	$44.94	$411.79	$755.80	$21,677.12
5 to <10 years old (n = 2)	Mean	$497.41	$11,245.62	$25,815.75	$11.23	$121.91	$405.76	$38,097.68
	SD	$502.46	$15,127.65	$1,929.60	$0.00	$18.24	$179.96	$17,397.98
	25th IQR	$142.12	$548.76	$24,451.31	$11.23	$109.02	$278.50	$25,795.45
	Median	$497.41	$11,245.62	$25,815.75	$11.23	$121.91	$405.76	$38,097.68
	75th IQR	$852.70	$21,942.49	$27,180.18	$11.23	$134.81	$533.01	$50,399.91
10+ years old (n = 5)	Mean	$284.23	$424.98	$14,752.10	$16.96	$152.62	$433.19	$16,064.10
	SD	$207.17	$646.96	$27,521.97	$13.87	$181.55	$442.86	$27,898.32
	25th IQR	$142.12	$34.67	$1,069.79	$11.23	$19.23	$89.51	$2,558.94
	Median	$213.18	$149.79	$1,801.34	$11.23	$132.18	$299.82	$4,249.39
	75th IQR	$426.35	$350.25	$6,361.83	$28.62	$158.58	$735.60	$6,869.00
Total (n = 14)	Mean	$319.76	$3,881.79	$24,755.53	$35.08	$205.30	$411.15	$29,608.62
	SD	$211.81	$6,328.14	$47,539.56	$41.54	$154.04	$392.34	$48,351.61
	25th IQR	$142.12	$149.79	$1,801.34	$11.23	$109.02	$0.00	$4,249.39
	Median	$284.23	$1,053.80	$5,206.59	$25.89	$154.11	$329.66	$8,445.01
	75th IQR	$426.35	$4,172.80	$24,454.34	$44.94	$332.96	$735.60	$25,795.45

SD: Standard deviation; IQR: Interquartile range.

The statistics shown commonly exhibit a large dispersion and a bias to the right of the distribution, regardless of the age group and area of care and a spread enhanced by the number of patients in certain age groups. The use of general medication (analgesics, sedatives, cardiovascular support drugs, and treatment for the primary disease, among others) represented the largest percentage among the items that make up the total cost. The comparison of the average costs in the emergency room revealed that the 10 years and older age group represented the highest financial burden (Table 2), while children under one year represented the highest burden in the general ward (Table 3). Naturally, the highest financial burden is represented by the patients in the PICU, especially children under one year of age (Table 4).

The mean total costs resulting from adding the costs of the three hospital areas were $33,116 (SD: $93,768) for the youngest age-group, $26,752 (SD: $49,481) for the 1–5 age-group, $28,354 (SD $46,263) for the 5 to 10 group, and $49,674 (SD: $87,715) for the oldest age-group (Table 5). Therefore, the mean total cost all over the age groups was $32,290 (SD: $62,918). Since the sample of patients admitted to the PICU was small the corresponding cost was also little relevant. Although the 10+ age-group resulted with the highest total cost there was no evidence of statistically significant differences between age groups.

**Table 5:** Total costs over the three clinical areas (Emergency Room, General Ward and PICU).

Age groups	Mean	Standard deviation	Percentile 25th	Median	Percentile 75th
<1 years old, (n = 14)	$33,115.93	$93,768.54	$976.91	$3,344.19	$15,699.25
1 to <5 years old, (n = 50)	$26,752.12	$49,481.23	$3,355.04	$9,844.81	$25,777.77
5 to <10 years old, (n = 43)	$28,353.70	$46,262.77	$1,645.34	$9,579.71	$35,683.21
10+ years old, (n = 25)	$49,674.53	$87,715.48	$7,734.89	$13,577.35	$69,456.99
Total, (n = 132)	$32,290.16	$62,917.94	$2,442.48	$9,710.62	$29,535.67

From the specification tests developed as a first stage of the multivariate analysis the gamma distribution family models resulted with the lowest values for the Akaike (3,006.98) and Bayesian (3,009.86) criteria respectively, in comparison with the rest values evaluated (See Table 6). Therefore, we decided to use the GLM log gamma to assess the effect of the covariables on the total influenza cost.

**Table 6:** Results of specifications tests.

GLM	N	AIC	BIC
Log gamma	132	3006.985	3009.867
Sqrt gamma	132	3006.985	3009.867
Log Gaussian	132	3292.687	3295.57
Sqrt gaussian	132	3292.687	3295.57
Log poisson	132	8560218	8560221
Sqrt poisson	132	8560218	8560221

Table 7 show the GLM results that were adjusted to assess the effect of the covariables on the sum of the total costs for the three hospital areas. As mentioned previously, the model was specified with a logarithmic link function assuming a gamma distribution family, which implies that the variance of the cost is proportional to the square mean. The coefficients for the model are shown in the first place; however, in econometrics it is preferable to interpret the coefficients in the terms of the marginal effects or discrete changes. The marginal effects are calculated when a predictor is continuous, while the discrete changes are used for categorical predictors. The Table 7 shows said values with the header dy/dx. Interpreting the effects that resulted statistically significant at a 5% alpha level, we find that, on average, the total cost is reduced in under 24,000 dollars for the group of children under one years versus the group of children aged 10 and above (reference category). It is important to highlight that the cost increases, on average, slightly over 10,000 dollars for patients with comorbidities, which would reflect the excess cost due to the fact that influenza presents on patients who are treated for in a third level institution. This would explain the high calculated cost for patients with cancer and bone marrow transplant compared with those who suffer from any comorbidity. Finally, we see that for each day in the hospital, the average increase of the cost is of 887 dollars; however, staying at the PICU was not statistically significant for a 5% alpha level, most likely due to the minimal number of patients in this category.

**Table 7:** GLM log gamma results for assessing the effect of different covariables on the new cost.

Total_cost_USD	Coefficient	Marginal or discrete effects (dy/dx)	P value	Confidence intervals (95%) for the marginal or discrete effects	
Men	0.150	$3,339.35	0.494	-$6,231.50	$12,910.20
Age group					
<1 year	-0.989	-$24,330.22	0.049	-$48,520.41	-$140.03
1 a <5 years	-0.573	-$16,896.88	0.174	-$41,283.09	$7,489.33
5 a <10 years	-0.690	-$19,309.92	0.082	-$41,037.42	$2,417.59
Comorbidities	0.622	$10,688.14	0.024	$1,424.17	$19,952.12
Allergic illness	2.142	$140,947.50	0.089	-$21,300.49	$303,195.60
Kidney disease	1.366	$57,193.58	0.250	-$40,301.02	$154,688.20
Neuromuscular disease	0.939	$29,023.14	0.271	-$22,634.96	$80,681.24
Neoplasia	1.428	$46,114.71	0.001	$19,211.41	$73,018.01
HSCT	1.780	$107,650.20	0.001	$43,065.91	$172,234.50
Death	-0.047	-$1,036.29	0.947	-$31,783.30	$29,710.72
Total hospitalization days	0.040	$887.49	0.012	$194.33	$1,580.66
Admitted to PICU	0.785	$24,582.59	0.085	-$3,368.56	$52,533.74
_cons	8.530				

HSCT: Hematopoietic stem cell transplantation; PICU: Pediatric Intensive Care Unit.

**Fig 2:**
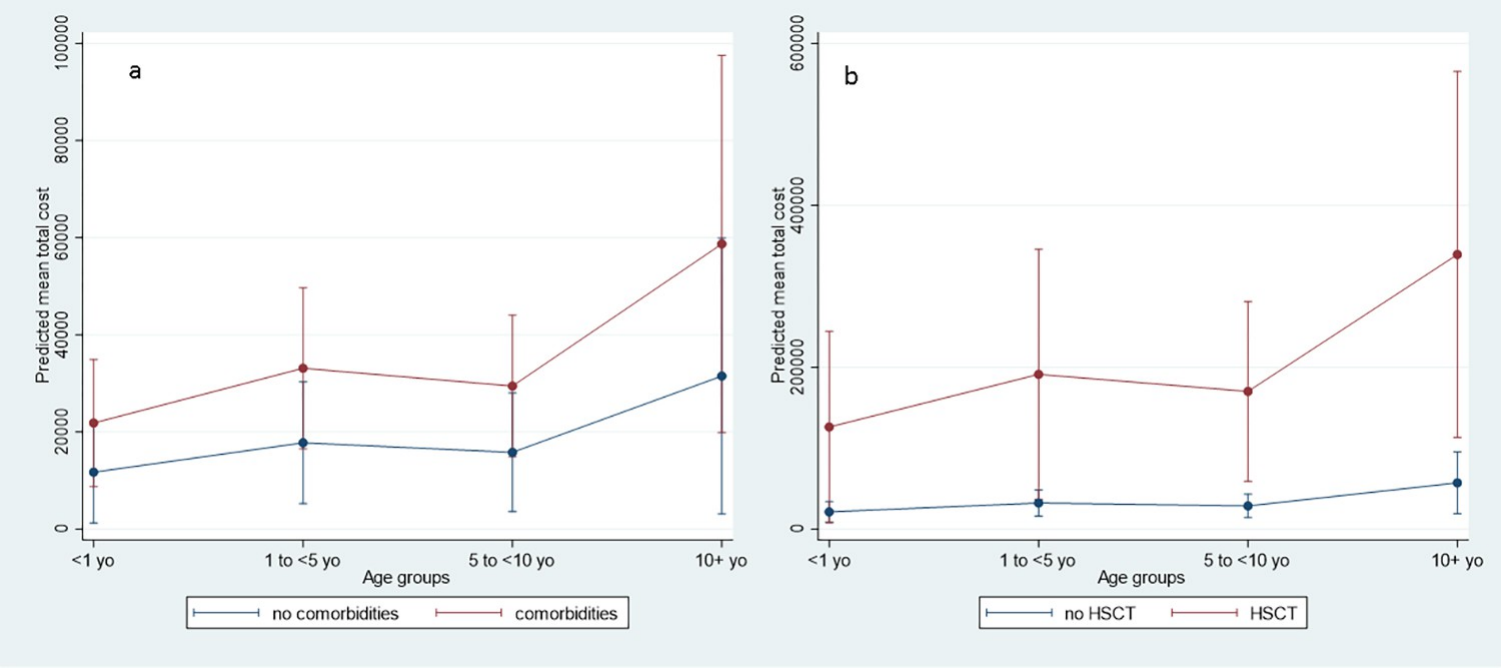
Total costs by age group for the presence of comorbidities (section a) and bone marrow transplant (section b).

**Fig 3:**
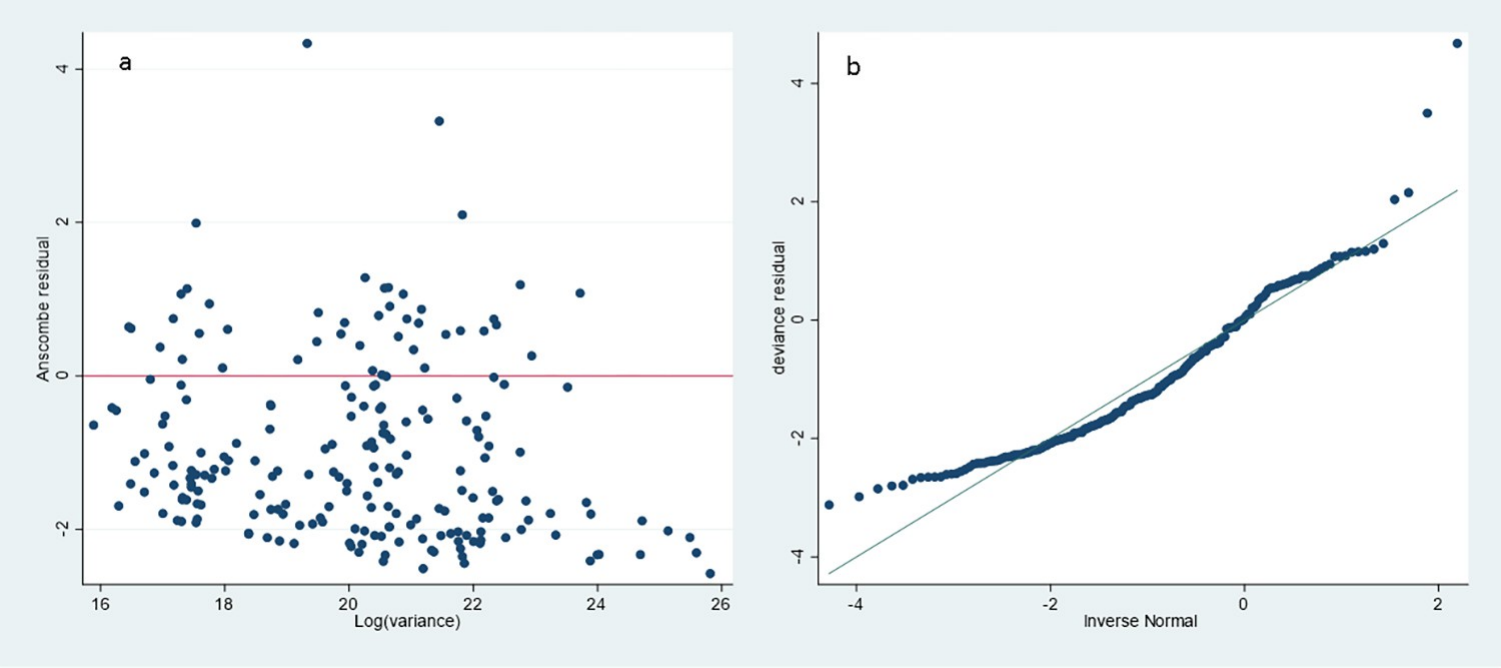
Anscombe (section a) and deviance (section b) residuals to assess the model adjustment.

Figs 2 and 3 shows different results for the GLM. Fig 2 section A shows the total costs calculated by age group and for the presence of comorbidities, while section B shows the total costs calculated by age group and bone marrow transplant. Fig 3 show the Anscombe residuals and the deviation in order to assess the model adjustment, which is acceptable for a non-linear model of this type.

## Discussion

This is the first study on the costs resulting from hospitalizations for influenza in Mexico by micro costing method, and one of the few Latin American studies on the topic. We must not forget that most of the patients included in this study had a primary disease besides influenza; therefore, the calculated costs would not reflect the economic burden of influenza exclusively. A prospective matched case-control design would be necessary to estimate the attributable cost of influenza. However, secondary pediatric hospitals in Mexico do not routinely perform the laboratory test to diagnose viral infections, for this reason, our study has the strength of having the influenza diagnosis for all the patients included despite being a retrospective study. Therefore, our results only reflect the cost of the care in patients whose admittance reason was influenza in the presence of comorbidities, treated for in a tertiary hospital. For this reason, the high calculated costs reflect, in turn, the quality of the attention and the therapeutic management patterns in an institution that provides attention to complex diseases. Our results are consistent with other reports that highlight hospitalizations due to influenza as one of the highest economic burden in Latin America. In Colombia, the median SARI cost was reported as $1826.00 USD, while in Mexico the median was $9710 USD (Table 5) [9]. When comparing the costs by the place of hospitalization, as in Colombia, the highest costs was for the patients who required a PICU. However, in other parts of the world, the costs are importantly different. In low-income countries such as Kenya, the calculated cost per episode per hospitalized subject was of $117 USD [10]; a prospective cohort in Thailand calculated the cost for children hospitalized with influenza; however, only 9 children without comorbidities and 9 with high-risk influenza complication comorbidities were hospitalized. The calculated cost was of $232 USD and $318 USD, respectively [11]. None of the studies analyzed the covariables that explained the increased costs. Our data is similar to those reported in the United States, where the median costs for children under 1 year was $5211.68 USD and for children aged 1 to 17 $7346.58 USD [12]. In our study focusing mainly on children with comorbidities, the cost was lower on children under 1 year of age, and higher in the rest of the pediatric aged children (Table 5), however no statistically significant differences were founded. Maybe the body weight was the main cost driver associated to the drug consumption that could explain the highest total cost of the 10+ age-group, as other factors were not available in our data. These costs must be interpreted in the scenario of a reference hospital where there is a possibility to treat the patient mostly with support measures, including drug products, antibiotics, and procedures that reflect a low hospital mortality (1.51%) even when dealing with complex patients [9–12].

Among the limitations of our study we can refer to the type of patients receiving specialized care at a tertiary hospital like ours, since they are significantly different from the patients attended at other healthcare institutions. For example, the average hospital stay was longer in our study than in Spain (mean 17.4 vs 9.9 days) [13], which the median was similar to those reported in Catalonia (6 days) [14]. Furthermore, the largest part of our population has comorbidities. Even in clinical trials in children, the rate of children with comorbidities is not as large; the largest rate of children with comorbidities was 30% in Spain and 39% in the USA [13–15], however, in our econometric model, the inclusion of the indicator variable for comorbidities allows us to identify the economic weight of this factor, which was up to $ 19,000 USD (Table 7).

Another limitation of this study was a reduced sample size for a variable that shows great variability, especially on the patients who were admitted to the PICU. However, in spite of this limitation, it was possible to adjust an adequate model for this type of response, which allowed us to identify the influenza cost predictors in a population with special characteristics, with pediatric patients that are treated for in a tertiary hospital since their diseases are highly complex, which by itself represents an important financial burden for the hospital. These results are relevant at a moment such as the present, when the COVID-19 pandemic has significantly affected the finances of healthcare institutions since the risk of an influenza syndemic can further compromise public institution budgets.

Within the disease burden, our data correspond to the tip of the pyramid which represents the most severe cases, in children with complex diseases and in a reference hospital, this gives us an idea from the impact of influenza on an economic point of view for the country and strengthens the need for more exact cost-effectiveness studies for new vaccines or new treatments. A cost-effectiveness study for expanding vaccination in Mexico was cost-saving even when the costs were calculated by assuming different scenarios, using costs reported by different health institutions in Mexico without a clear methodology of how the use of resources was estimated [16]. This might have underestimated the real cost of the resources, another weakness of this study is that it does not consider herd immunity, new studies must be performed for these variables in order to discuss, with the people in charge of making decisions, the expansion of the vaccination against influenza to school-age children, especially now that we are going through the COVID-19 pandemic. With a potential syndemic with influenza, and less than 50% of the population vaccinated against COVID-19, it is especially important to have these discussions in order to maximize the collective benefit of vaccination within the national vaccination schedule.

To conclude, this is the first study on the use of resources by micro-costs in Mexico with the largest N in Latin America, and that performs an econometric analysis. Along with calculating the costs, we were able to determine that comorbidities (neoplasia and HSCT), along with the hospital stay duration, are the largest determiners of the cost increase (Table 7 and Fig 2). It is necessary to boost vaccination programs in high-risk children who could benefit from immunization for their family members, along with the expansion of the recommendation of vaccination for school-aged children. New cost-effectiveness studies that aim to expand the immunization in school-age children which include the assessment of herd immunity are needed.

## Supporting information

S1 File (XLS)

S2 File (DOCX)
