# Supplementary material for: Economic issues of Severe Acute Respiratory Infections for influenza in Mexican children attended in a tertiary public hospital
Source: PLoS One. 2022 Sep 9;17(9):e0273923. doi: 10.1371/journal.pone.0273923 (PMC9462801; doi:10.1371/journal.pone.0273923)
Supplement: S2 File — (DOCX) [file pone.0273923.s002.docx]

Enero de 2018

Carga económica de la infección respiratoria aguda grave por influenza en niños de un hospital de tercer nivel de la Ciudad de México

[Subtítulo del documento]

Rodolfo Norberto Jiménez Juárez Sarbelio Moreno Espinosa

Ana Estela Gamiño Arroyo

Alfonso Reyes López

Investigadores

INDICE

[RESUMEN 2](#_Toc505218427)

[INTRODUCCIÓN 3](#_Toc505218428)

[Evaluación de costos. 6](#_Toc505218429)

[PLANTEAMIENTO DEL PROBLEMA 10](#_Toc505218430)

[JUSTIFICACIÓN 11](#_Toc505218431)

[OBJETIVO GENERAL 12](#_Toc505218432)

[OBJETIVOS ESPECIFICOS 12](#_Toc505218433)

[HIPÓTESIS DE TRABAJO 12](#_Toc505218434)

[METODOLOGÍA 13](#_Toc505218435)

[*Diseño* 13](#_Toc505218436)

[*Población* 13](#_Toc505218437)

[*Criterios de inclusión* 14](#_Toc505218438)

[*Criterios de exclusión* 14](#_Toc505218439)

[*Proceso general para estimar la carga económica de la IRAG por influenza* 14](#_Toc505218440)

[*Etapa 1. Identificación de recursos* 16](#_Toc505218441)

[*Etapa 2. Planeación del marco de muestreo* 16](#_Toc505218442)

[Etapa 3. Medición de la utilización de recursos hospitalarios 17](#_Toc505218443)

[Etapa 4. Determinación de costos unitarios 18](#_Toc505218444)

[Etapa 5. Estimación de los gastos de bolsillo y costos indirectos 18](#_Toc505218445)

[*Análisis de los costos por paciente* 18](#_Toc505218446)

[*Variables* 19](#_Toc505218447)

[Factibilidad. 19](#_Toc505218448)

[Consideraciones Éticas. 20](#_Toc505218449)

[BIOSEGURIDAD 21](#_Toc505218450)

## RESUMEN

Antecedentes. Las infecciones por virus de la influenza se presentan cada año, afectando aproximadamente al 20% de los niños de todo el mundo. Dentro de las hospitalizaciones por infección respiratoria aguda aproximadamente el 10% son ocasionadas por el virus de la influenza, sin embargo, a nivel local y regional no hay estudios sobre la carga de la enfermedad en su sentido más amplio, y los pocos estudios a nivel global que hay, se centran en la epidemiología que son solo una de las partes de la carga de enfermedad.

Objetivo. Determinar la carga económica de la infección respiratoria aguda grave por influenza en niños.

Metodología. Estudio observacional, descriptivo, ambispectivo, la parte prospectiva se requiere para medir costos indirectos. Estudio con base de revisión de expedientes de niños hospitalizados al menos 24 hrs. en el HIMFG por infección respiratoria aguda grave y que tengan PCR multiplex positivo para influenza. Sujetos elegibles Infección respiratoria aguda grave por virus de influenza. Evaluación de uso de recursos: Microcosteo a partir de expedientes clínicos, evaluación de costos indirectos a partir de encuesta a cuidadores.

Plan de análisis de los datos. Se estimará el costo de la enfermedad global y se dividirá en costos directos e indirectos; los costos directos serán subdivididos en costos secundarios a hospitalización, medicamentos, exámenes de laboratorio, exámenes de gabinete, procedimientos; para estimar costos indirectos se utilizará la aproximación de. Se estimarán medidas de tendencia central y de dispersión, los costos serán expresados en pesos mexicanos.

Etica. Estudio sin riesgo dado que solo se revisarán expedientes, y la encuesta que se aplicara no trata aspectos sensitivos de la conducta del cuidador.

Bioseguridad. No tiene implicaciones de bioseguridad, el proyecto no contempla trabajar con muestras de sujetos de investigación, se revisarán los resultados de las pruebas que se toman en la atención clínica estándar.

## INTRODUCCIÓN

El virus de la influenza es uno de los patógenos respiratorios más importantes en todo el mundo. Cada año, las epidemias de influenza afectan a aproximadamente el 5% de los adultos y el 20% de los niños de todo el mundo. ^1^ Aunque la influenza provoca infecciones autolimitadas leves en la mayoría de los pacientes, varios estudios han demostrado en la última década que el virus tiene un impacto significativo en la demanda de servicios de salud por la población pediátrica [2-3]. La influenza no sólo repercute en la tasa de hospitalización, sino que también aumenta el número de visitas médicas y el uso de antibióticos. Cada año entre 6 y 15 visitas médicas se atribuyen a la gripe por cada 100 niños. Además, se estima que, por cada 100 niños 3-9 tratamientos de antibióticos se prescriben por las infecciones de la influenza cada año [3]. Aunque la morbilidad y la mortalidad del virus de la influenza se han identificado como una carga importante tanto para los países en desarrollo como para los desarrollados, los datos de calidad que describen el impacto de las infecciones estacionales confirmadas por la influenza en México siguen siendo muy limitados.

México cuenta con un amplio programa de inmunizaciones que incluye la vacuna contra la influenza. Las recomendaciones actuales consideran a los niños de 6 a 60 meses de edad [4]. Las infecciones respiratorias agudas (IRA) son la principal causa de morbilidad reportada por la Secretaría de Salud de México en niños menores de 6 a 60 meses de edad. Se sabe que los niños menores de 60 meses tienen un mayor riesgo de infecciones respiratorias. Sin embargo, cada año durante la temporada de gripe, un número importante de hospitalizaciones secundarias a infecciones respiratorias agudas se presentan en niños de 5 a 10 añosde edad [5]. Los agentes virales son la etiología más común de las IRA entre la población pediátrica, incluyendo el virus sincicial respiratorio (RSV), virus parainfluenza, adenovirus, metapneumovirus humano y virus de la influenza A y B [6-8].

La estrategia principal de prevención de infecciones y enfermedades causadas por el virus de la influenza es la inmunización activa. En México existe un extenso programa de vacunación contra la influenza, que considera 100% niños de 6 meses a 59 meses de edad y 100% de adultos mayores de 60 años de edad. Sin embargo, con respecto a los niños entre 5 y <10 años de edad, sólo la vacunación se considera para aquellos con factores de riesgo [4]. Las vacunas utilizadas en México para prevenir la gripe son vacunas de virus inactivadas. Su eficacia oscila entre el 60 y el 90% dependiendo de la concordancia entre la circulación viral, la edad de los sujetos y sus condiciones médicas [10].

Los casos de enfermedad tipo influenza (ETI) de acuerdo a la OMS se definen como pacientes con una infección respiratoria aguda:

- con historia de fiebre o fiebre medida de ≥ 38 °C;
- con tos;
- con inicio en los diez días precedentes;

Los casos de infección respiratoria aguda grave (IRAG) de acuerdo a la OMS se definen como pacientes con una infección respiratoria aguda:

- con historia de fiebre o fiebre medida de ≥ 38 °C;
- con tos;
- con inicio en los diez días precedentes;
- y que requieren ser hospitalizados [9].

En una revisión sistemática de carga global de la enfermedad de 1982 a 2012 se encontró que la influenza se asoció con un 10% (95%, IC 8-11%) de hospitalizaciones en niños menores de 18 años en todo el mundo. En promedio, se estimó que la influenza resultó en aproximadamente 374,000 hospitalizaciones en niños menores de 1 año, de los cuales 228,000 en menores de 6 meses y 870,000 en niños menores de 5 años anualmente. La influenza se asoció a una tasa de hospitalización tres veces mayor en países en desarrollo que en países industrializados (150/100,000/año contra 48/100,000) [11]. En Francia se estudió el impacto económico de la influenza B, se estudiaron a 201 sujetos y se estimó los costos directos de atención, tanto en pacientes ambulatorios como hospitalizados, aunque no detallan el porcentaje de pacientes hospitalizados y la estimación de los costos global. Los autores reportan un costo de $70€ (IC95% 0.4 – 139.6) para niños de menos de 4 años y de $50€ (IC95% 12.0 – 88.0) para niños 5 años o mayores. [12]

En una revisión sistemática realizado en la Región Mediterránea Oriental que comprende 22 países de diferentes ingresos per cápita, de 1990 a 2015. Este estudio utilizó análisis de meta-regresión Bayesiano para estimar la carga de la enfermedad de las infecciones de vías respiratorias bajas (IVRB), basado en muerte, años de vida perdidos (YLL), años de vida viviendo con discapacidad y años de vida ajustados por discapacidad (DALYs siglas en inglés disability-adjusted life year). Se encontró que las IVRB son la tercera causa de muerte en todos los grupos de edad en 2015, siendo la tasa y número de muertes mayor en los niños menores de 5 años. Además, las IVRB son la cuarta causa de DALYs causando 11,098,243 (95%, 9,857,095-12,396,566) DALYs y 191,114 (95%, 170,934-210,705) muertes. Las IVRB son la tercera causa de YLL en 2015. De acuerdo con la etiología, *S. pneumoniae* tuvo la mayor tasa de mortalidad (16.6 por 100,000, 95% 10.0-22.9). Seguido de *Haemophilus influenzae* tipo b (1.1 por 100,000, 95% 0.9-2.3) y el virus de la influenza (0.8 por 100,000, 95% 0.5-1.2). Este estudio parte del estudio de Carga de la enfermedad, daños y factores de riesgo 2015 (Global Burden of Diseases, Injuries, and Risk Factors 2015 que incluyó 195 países, 21 regiones y 7 súper-regiones para estudiar 315 enfermedades y lesiones y 79 factores de riesgo). [13]

Una revisión sistemática anterior de la literatura de la carga económica y de la evaluación económica de estacional la influenza encontró un total de 140 estudios en todo el mundo, de los cuales 39 estudios (28%) fueron costo-de-enfermedad estudios [8]. Dos artículos no declararon su ámbito de aplicación (es decir, la configuración) o la perspectiva que normalmente se ha hecho en un informe apropiado de los estudios del coste [9]. 32 (82%) de los 39 estudios se realizaron en los países de ingresos altos. Perspectiva social – es decir, un análisis que incluye todos los costos y beneficios de una intervención sanitaria, independientemente de quién pague por ella, se utilizó comúnmente (21 estudios, 54%). Entre los 24 estudios que reportan un horizonte temporal, 15 estudios lo especificaron como un horizonte temporal de 1 año. Nueve estudios cuantificaron los costos directos médicos, no médicos e indirectos, mientras que ocho estudios incluían sólo gastos médicos directos y no médicos directos. Catorce estudios cuantificados indirectos costos. Diecisiete estudios estimaron la carga económica sobre la base de la estacionalidad confirmada por laboratorio casos de influenza, mientras que 21 estudios lo estimaron usando casos clínicamente diagnosticados. Una sistemática reciente el examen de la evaluación económica de las vacunas contra la gripe en PIMB indicó que la los datos usados no-laboratorio confirmaron sari o ETI. No se utilizaron costes indirectos en estos análisis [10].

Por último, ningún estudio tuvo en cuenta los costos de la atención informal entre la atención médica no atendida (es decir, los costos incurridos por personas que no buscan atención en centros de salud formales).

### Evaluación de costos.

Los costos relevantes que considerar en una evaluación económica dependen de la perspectiva que asume la evaluación. Los pacientes, prestadores y financistas, tienen perspectivas distintas, porque trasladan costos de forma diferente. Para un prestador los costos directos son los relevantes. El paciente, dado que los seguros financian la atención, considera lo contrario. Los costos directos, se asocian con los recursos propios de los sistemas de salud, mientras que los costos indirectos se relacionan con el valor del tiempo que tiene que invertir el paciente en una intervención (traslado, espera, recuperación, etc.), asociado al salario y a su productividad. El costo social se refiere a los costos que afectan a todos y cada uno de los miembros de la sociedad.

El costo corresponde al valor del consumo de recursos en que se incurre para generar un servicio, el cual puede ser económico o financiero. Cuando es financiero, el costo implica un desembolso monetario, cosa que no necesariamente ocurre cuando es económico. Los costos tienen dos componentes: el consumo físico de recursos asociado al proceso de provisión y la asignación de un valor a este consumo físico. Cuando expresamos el consumo de recursos en términos unitarios podemos hablar de precio.

El precio que habría que utilizar para valorar los recursos es el costo de oportunidad. Técnicamente, su cálculo implica conocer la función de bienestar social, cosa que la economía no ha podido realizar. Bajo ciertos supuestos –equilibrio competitivo y aceptar la distribución de ingresos– los precios de mercado de los recursos son buenos estimadores de los costos de oportunidad. En los sistemas de salud los precios no son buenos estimadores: están distorsionados por fallas de mercado, existen terceros pagadores y/o intervención estatal, desequilibrio o inexistencia de mercados y precios inestables e impredecibles.

El análisis microeconómico, ayuda a entender y diferenciar el comportamiento de los costos. Los costos totales, consideran el total de recursos que se consumen para generar la producción. Pueden descomponerse según el tipo de recursos productivo (trabajo, insumos, capital, etc.) o según su asociación con el volumen de producción. Los costos fijos no varían con esta última. Los costos de capital – las camas, equipamiento, administración, etc.– son costos fijos. Los costos variables –trabajo, insumos, medicamentos, etc.–, están relacionados con los niveles de producción. Esta distinción entre costos fijos y variables es válida mientras no se altere la capacidad productiva total de la unidad. Esto se denomina corto plazo y no está asociado a un período temporal específico. En un horizonte que permita el redimensionamiento total de la actividad –conocido como largo plazo–, todos los recursos son variables.

Los costos totales y costos unitarios están asociados. Los costos unitarios se refieren al costo de producir una unidad, entre ellos los costos medios y los marginales. En el corto plazo, los costos unitarios son directamente proporcionales al pago efectuado para disponer del recurso e inversamente proporcionales a su productividad. Así, para un salario dado, mientras mayor sea la productividad del trabajo –el principal recurso de los sistemas de salud– menor es el costo unitario de producción.

La estimación de costos supone, además de definir correctamente el problema que se quiere abordar –objeto del costeo– y una descripción detallada del proceso asociado al servicio que se quiere costear, otras tres etapas: Identificar el consumo de recursos asociados a las intervenciones; cuantificar el consumo físico de recursos; y, valorizar el consumo de recursos.

En la identificación del uso de recursos es conveniente distinguir: i) Recursos de atención (médicos, insumos, fármacos, etc.) requeridos directamente en la intervención; ii) Recursos de atención que en el futuro son inducidos o son evitados por la intervención; iii) Tiempo del paciente incluyendo los cambios en la productividad; y, iv) Tiempo de cuidadores del paciente y costos informales asociados. Dependiendo de la perspectiva de la evaluación uno u otro componente podrán incorporarse, incluyendo todos en la perspectiva social.

Es importante el horizonte temporal de la evaluación: El período donde la intervención genera efectos. En la medida que las intervenciones no alteren sustantivamente la capacidad de producción y no impliquen la posibilidad de sustituciones importantes entre factores –por ejemplo, entre trabajo y capital– es aceptable trabajar con costos marginales de corto plazo.

La cuantificación del uso de recursos puede ser prospectiva o retrospectiva. Bajo la primera, se preparan, como parte de los estudios clínicos aleatorizados, los formularios que recabarán información de consumo de recursos asociados a los protocolos de atención. El retrospectivo –usar las fuentes de registro rutinario de los establecimientos de salud–, es el más utilizado. El esfuerzo necesario para estimar los costos debe ser proporcional a la importancia de los mismos. No conviene invertir tiempo y recursos en costos que son irrelevantes en la decisión.

La base de cálculo del consumo de recursos puede tener distinto nivel de detalle o bases de costeo. En el extremo, el método que entrega mayor precisión está el costeo de cada una de las atenciones, denominado micro-costeo. Se identifican actividades (consulta médica, los exámenes de apoyo diagnóstico, los días cama, etc.) para cada una de las fases de la intervención para cada tipo de paciente.

El costeo sobre la base de una canasta estandarizada de prestaciones, tipo GRD, en el cual se toma el costo de resolución de un caso para un paciente promedio. En este caso, no es necesario identificar cada una de las actividades que involucra la intervención, sino sólo el conjunto estandarizado de intervenciones que supone la resolución del problema.

Menos preciso es considerar el costo promedio diario asociado al tratamiento en cada categoría de enfermedad. Este método corresponde a un híbrido entre el método anterior y el método del costo promedio diario de atención, donde se realiza un esfuerzo por asociar el consumo de recursos a un tipo de paciente.

Finalmente, el método más impreciso es tomar el valor que comúnmente está disponible en los sistemas de salud, es decir, el costo promedio diario de atención sobre todas las categorías de pacientes, por ejemplo, el valor del egreso medio.

La valorización consiste en determinar el “precio” que hay que pagar por los recursos, tomando en cuenta la perspectiva de la evaluación. Los financieros optan por ponderar las atenciones por los precios de transferencia que utilizan para financiar las actividades de salud. Esto se fundamenta, en que los seguros pueden acceder a estas actividades a través de diversos prestadores.

Lo anterior no coincide con la perspectiva del prestador, el cual incurre en costos para proveer la intervención que pueden estar por sobre o bajo el precio del seguro. Dado su mayor tamaño financiero, los seguros imponen precios “monopsónicos”, menores a los que resultarían de un equilibrio competitivo. Desde una perspectiva social, el problema es complejo y no tiene fácil solución.

Los costos no se incurren en un mismo momento, sino que se distribuyen en el tiempo. La sociedad no valora igual disponer de recursos para el consumo hoy, que disponer de los mismos en el futuro. Esto se conoce como preferencia intertemporal. Está sesgada hacia una mayor valoración de los sucesos que ocurren en el presente. La medida en que la sociedad está dispuesta a sacrificar consumo presente por consumo futuro se conoce como tasa de preferencia intertemporal y sirve para darle valor al desplazamiento de recursos de un período a otro.

Figura 1. Unión entre la presentación clínica de influenza y la carga económica de la enfermedad.

La pandemia de A(H1N1) del 2009 destacó la necesidad de recopilar información sobre la gravedad de la enfermedad de una manera estandarizada y de contar con datos históricos para que los países puedan evaluar su situación actual en el contexto de las temporadas de influenza anteriores, por lo que la Organización Panamericana de la Salud (PAHO) ha establecido guías específicas para la vigilancia. El presente estudio tendría el alcance de vigilancia basada en indicadores.

El propósito de este estudio es proporcionar información de la carga económica de los niños con infección respiratoria aguda grave secundaria al virus de la influenza, desde un enfoque de carga de la enfermedad basada en vigilancia de laboratorio (Fig. 1).

## PLANTEAMIENTO DEL PROBLEMA

El virus de la influenza tiene la capacidad de tener variaciones antigénicas qué ocasionan pandemias que pueden derivar en una gran mortalidad, es por ello que a partir de la pandemia de 2009 los países de América Latina han fortalecido sus sistemas de vigilancia epidemiológica con el objetivo de detectar nuevos subtipos del virus de influenza.^2^ En México el Boletín de Información Estadística 2014, coloca a neumonía e Influenza como la séptima causa de mortalidad general, con una tasa de 17.1/100,000 personas y la décima causa de egresos hospitalarios.^3^ Durante el inicio de temporada de influenza 2017-2018, la Dirección General de Epidemiología reporto el 28 de diciembre de 2017, 534 infecciones confirmadas por influenza y 7 defunciones, el subtipo que ha predominado es AH3N2.^4^ En México la vigilancia de la Influenza se realiza bajo las estrategias de tipo centinela y sindromática avalada por la OMS, con información proveniente de 541 Unidades de Salud Monitoras de Influenza (USMI), mediante los criterios de ETI/IRAG, y confirmación por laboratorio para conocer la positividad de casos y su subtipificación. Como se puede observar hay evidencia de la carga epidemiológica de la enfermedad en la población general, sin embargo, no está delimitada la carga económica, incluso la OMS desarrollo un manual con una metodología detallada para llevar a cabo la evaluación de la carga económica de la influenza estacional, para que cuando esta información este disponible en diferentes partes del mundo pueda ser comparable.

## JUSTIFICACIÓN

Los gobiernos nacionales requieren datos sobre la carga económica de la influenza en sus países para tomar decisiones informadas y basadas en evidencias a fin de poder asignar óptimamente los recursos limitados y priorizar las intervenciones en el sector salud. Una revisión sistemática reciente sobre la carga económica de la influenza en países de ingresos bajos y medianos (PIBM) sugiere que la información es escasa o incompleta y que existe una falta de métodos estandarizados para las estimaciones de costos. Dada la escasez de información acerca de la carga económica de la influenza estacional en los PIBM, la OMS desarrollo un manual para la estimación de la carga económica de la influenza estacional para apoyar la estandarización de las estimaciones entre países. Su objetivo es facilitar que los funcionarios de estos países lleven a cabo estudios que evalúen la carga económica de esta enfermedad para apoyar a los responsables de las políticas de salud para decidir acerca de la introducción de una vacuna contra la influenza, particularmente si los recursos limitados disponibles deben ser asignados para garantizar la priorización óptima de varias intervenciones de salud y apoyar en la toma de decisiones sobre estrategias de vacunación complementaria o ampliación de los grupos de vacunación. En dicho Manual se señala que la estimación del costo total de la influenza debe incluir tres componentes: los costos médicos directos, los costos no médicos directos y los costos indirectos.

## OBJETIVO GENERAL

Estimar la carga económica de la infección respiratoria aguda grave (IRAG) por influenza en niños de un hospital de tercer nivel.

## OBJETIVOS ESPECIFICOS

1. Estimar los costos médicos directos de la IRAG por influenza en niños
2. Estimar los costos no médicos directos de la IRAG por influenza en niños
3. Estimar los costos indirectos de la IRAG por influenza en niños

## HIPÓTESIS DE TRABAJO

Nuestra hipótesis de trabajo se centra en la estimación del costo promedio total de la enfermedad en cuestión. Por lo tanto, apoyándonos en un artículo de revisión [REF] acerca de la carga económica de la influenza en países de ingresos medios y bajos la literatura, asumimos que nuestra estimación estará cercana al costo reportado para dos países latinoamericanos, que fue de 525 dólares por paciente.

## METODOLOGÍA

### *Diseño*

Estudio observacional, descriptivo, ambispectivo. En la figura 1 se muestra un esquema del diseño del estudio para dejar claro el tipo de información que será obtenida y las estimaciones que se realizarán en cada etapa.

Figura 1. Tipo de información que se recolectara en cada etapa del estudio.


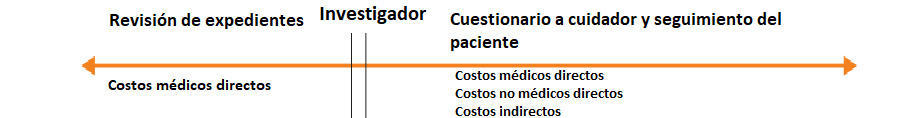


### *Población*

Pacientes atendidos en el Hospital Infantil de México Federico Gómez. La población de estudio será los niños hospitalizados con Infección Respiratoria Aguda Grave. Se seleccionaran a partir de la base de datos del laboratorio de biología molecular, se utilizará el registro de PCR multiplex para detección de virus respiratorios del 1 de octubre 2013 al 30 de mayo de 2019 (temporadas de virus respiratorios 2013-2014 a 2018-2019).Se revisaran todos los expedientes de los sujetos a los que se les tomó muestra para la detección de virus respiratorios por micro-arreglos, se estima que se tomaron muestras respiratorias al menos a 5000 sujetos en el tiempo de duración del estudio, en estos sujetos serán evaluados los criterios de selección para ser reclutados.

Aunque podríamos comenzar la evaluación de los expedientes en enero de 2017, este estudio será retrolectivo para la evaluación de desenlace clínicos y costos directos, sin embargo, para la evaluación de costos indirectos, la evaluación será a partir de los casos incidentes, en cuanto se autorice la realización del estudio por parte de los comités de investigación, ética y bioseguridad, se aplicará un cuestionario solo a los sujetos con IRAG por influenza.

Figura 2. Diagrama para la delimitación de la población de estudio.

### *Criterios de inclusión*

1. Menor de 18 años
2. Infección respiratoria aguda grave por influenza
3. Expediente disponible

### *Criterios de exclusión*

1. Infección respiratoria grave por influenza de adquisición nosocomial

### *Proceso general para estimar la carga económica de la IRAG por influenza*

La estimación de la carga económica (total) de la IRAG por influenza se entiende como la suma de los costos médicos directos, los costos no médicos directos y los costos indirectos; tal y como se esquematiza en la figura ##. Esta sección describe el proceso general recomendado por la OMS para estimar la carga económica de la IRAG por influenza, que se compone de cinco etapas (ver tabla ##), las cuales pueden ser categorizadas en tres actividades principales:

- Identificación de recursos
- Recolección-medición de datos
- Valoración de costos

El primer paso es identificar todos los recursos necesarios para tratar un episodio de IRAG por influenza. El segundo paso es planificar el marco de muestreo y la recopilación de datos. El tercer paso implica medir el uso de recursos utilizados en el tratamiento de los casos de IRAG por influenza confirmados por laboratorio. El cuarto paso consiste en describir cómo son valorados los costos, lo cual permite conocer la dirección en la determinación de los costos unitarios. El quinto paso busca estimar los gastos de bolsillo (médicos directos y no médicos directos) y los costos indirectos de los casos atendidos.

Figura 3. Alcance de la estimación de la carga económica para la IRAG por influenza

Tabla 1. Proceso de estimación de la carga económica de la IRAG por influenza

| Etapa | Descripción | Detalles |
| --- | --- | --- |
| 1 | Identificación de los recursos requeridos | Todos los recursos utilizados en el episodio de influenza |
| 2 | Planeación del marco de muestreo | Delimitación de la población de estudio |
| 3 | Medición de la utilización de recursos hospitalarios | Costos médicos directos |
| 4 | Determinación de costos unitarios |  |
| 5 | Estimación de los gastos de bolsillo (en relación con la atención hospitalaria) y de los costos indirectos (pérdidas de productividad) | Costos médicos directos, no médicos directos y costos indirectos |

La carga económica de la influenza se estimará usando el enfoque de micro-costos, que consiste en la multiplicación de la cantidad de recursos utilizados (Q) por el costo unitario o precio (p). En seguida se describen detalladamente cada una de las etapas señaladas en la tabla 1.

### *Etapa 1. Identificación de recursos*

Esta primera etapa consistirá en identificar todos los recursos utilizados en el episodio de influenza, es decir, la cantidad o frecuencia de los siguientes recursos: medicamentos, insumos médicos, pruebas diagnósticas, días de estancia hospitalaria, traslados, horas o días ausentes del trabajo o pérdidas de productividad de cuidadores.

### *Etapa 2. Planeación del marco de muestreo*

Para probar la hipótesis de nuestro estudio realizamos el cálculo del tamaño muestral utilizando la fórmula para estimar una media, asumiendo una población finita, ya que, de acuerdo con los anuarios estadísticos de nuestro hospital, anualmente se hospitalizan en promedio 500 casos de IRAG por influenza. Así, la fórmula utilizada es la siguiente:

$$n=\frac{N*Z_{\propto}^{2}*S^{2}}{d^{2}*\left( N-1 \right)+Z_{\propto}^{2}*S^{2}}$$

Donde:

*N* es el tamaño de la población

$Z_{\propto}^{2}$ es el valor de la distribución normal estándar fijado en 1.96 para lograr un nivel de confianza o seguridad de 95%.

*d* es la precisión con que se desea estimar el parámetro de interés.

*S^2^* es la varianza de la distribución de la variable cuantitativa que se supone existe en la población.

De acuerdo con la fórmula antes señalada, todos los valores de los parámetros pueden ser determinados por el investigador, con excepción de la varianza del estimador a ser calculado, la cual fue derivada de los resultados del artículo de revisión mencionado en nuestra hipótesis de trabajo, donde planteamos que la carga económica de la IRAG por influenza en nuestro país sería cercana a 525 dólares por paciente; por lo tanto, asumimos que la desviación estándar de la distribución tendría el mismo valor (525 dólares), lo cual equivale a decir que el coeficiente de variación es del 100%. Este supuesto tiene lógica ya que se ha demostrado que la distribución de los costos de una enfermedad por lo regular exhibe gran dispersión. Finalmente, fijando la precisión de la estimación en 5 dólares, se obtuvo un tamaño de muestra de 150 pacientes nuevos a incluir para el diseño prospectivo del estudio. Para el diseño retrospectivo tendremos la posibilidad de revisar los 500 expedientes de los pacientes que se hospitalizan anualmente por influenza.

### Etapa 3. Medición de la utilización de recursos hospitalarios

Para la recolección de datos sobre los recursos utilizados durante la atención hospitalaria de los pacientes, se diseñarán formularios electrónicos con el programa Access (Microsoft®), con secciones específicas para aquellos pacientes que hubieran ingresado a la terapia intensiva. Se reclutará y capacitará a encuestadores con perfil de salud para la revisión de los expedientes clínicos y para la aplicación de encuestas a cuidadores, realizando pruebas piloto para garantizar el adecuado desenvolvimiento durante el levantamiento de la información. El trabajo de los encuestadores será supervisado por los investigadores responsables con ayuda de algún médico coordinador.

### Etapa 4. Determinación de costos unitarios

Los costos unitarios o precios de los recursos utilizados para la atención hospitalaria serán obtenidos de los tabuladores de cuotas del HIMFG, específicamente los valores correspondientes al nivel socioeconómico más alto (nivel 6), que en teoría refleja el costo real de cada recurso.

### Etapa 5. Estimación de los gastos de bolsillo y costos indirectos

Se aplicará un cuestionario semi estructurado (ver anexo) a los cuidadores de los pacientes, previa firma del consentimiento informado. El cuestionario será validado en la fase piloto del estudio, para lo cual será sometido al escrutinio de un grupo de expertos quienes darán su opinión en cuanto a la validez de contenido, posteriormente se utilizará el procedimiento conocido como “test-retest” para evaluar confiabilidad del cuestionario. En lo que respecta a la estimación de los costos indirectos (pérdidas de productividad), para valorar en términos monetarios el tiempo perdido de los cuidadores se utilizará el enfoque del capital humano, es decir, se obtendrá información acerca de los ingresos actuales de los cuidadores.

### *Análisis de los costos por paciente*

Para estimar los costos específicos por paciente, las cantidades de recursos se multiplicarán por sus respectivos costos unitarios, los cuales serán presentados como promedios y desviaciones estándar para los siguientes indicadores:

- Costo por episodio de hospitalización (costos médicos directos)
- Gastos de bolsillo por episodio de hospitalización (costos médicos directos y no médicos directos)
- Costo total del tratamiento por paciente, con y sin costos indirectos (pérdidas de productividad)

Adicionalmente los resultados de costos se presentarán en forma estratificada por:

1. Grupo de edad 0 a 2 años, 2 a 5 años y ˃5 años
2. Coinfección bacteriana, definida como los sujetos que al ingreso al hospital tuvieron detección de bacterias en hemocultivo o en aspirado traqueal el día de la intubación o consolidación en la Radiografía de tórax o procalcitonina >0.05 ng/ml al ingreso o Proteína C reactiva >9 mg/dl al ingreso
3. Coinfección viral, definida como la detección de uno o más virus, diferente(s) al virus de influenza en hisopado nasofaríngeo por la técnica de PCR multiplex o dos subtipos de virus de influenza
4. Gravedad, clasificada en los siguientes estratos: a) Sin Sepsis ni ventilación mecánica; b) Sepsis o ventilación mecánica; o c) Choque séptico
5. Comorbilidad, definido como la presencia de una enfermedad previa al inicio de la infección.

### *Variables*

La única variable que se medirá en el estudio es el costo de la enfermedad.

1. Costo total de la enfermedad.

Definición conceptual: Valor del consumo de recursos en que se incurre para generar un servicio, el cual puede ser económico o financiero

Definición operativa: Cantidad en pesos mexicanos para la atención del sujeto de estudio

Tipo de variable: Cuantitativa continua

Escala de medición: Pesos mexicanos

###

### Factibilidad.

El estudio es factible considerando que se tiene una base de datos con el registro del paciente y el archivo clínico guarda los expedientes al menos 5 años, sin embargo, uno de los problemas al que potencialmente nos enfrentaremos es que el paciente sin comorbilidades y solo estuvo solo en urgencias y su expediente está en el archivo de corta estancia, lo cual nos generaría un sesgo de selección atribuible al tipo de Instituto que somos, este sesgo se controlará en el análisis estadístico. Se cuenta con un presupuesto de fondos externos por parte de Sanofi-Pasteur (Iniciativa del investigador) para poder contratar personal para la revisión del total de los expedientes, traducción de (las) publicación(es) y pago por publicaciones.

## Consideraciones Éticas.

El actual proyecto de investigación se basa en todas aquellas normas y estándares éticos, legales y jurídicos con la finalidad de proveer el respeto y protección a su salud y derechos individuales a todo aquel que se haya incluido en el estudio. De acuerdo con el reglamento de la Ley General de Salud en Materia de Investigación para la Salud, calificamos a nuestro estudio como una investigación sin riesgo pues no se realiza ninguna intervención o modificación intencionada en variables fisiológicas, psicológicas y/o sociales de las personas que participan en el estudio, en el artículo 17 de la Ley General de Salud en Materia de Investigación, también se considera que la aplicación de cuestionarios en los que no se tratan aspectos sensitivos de su conducta. En este documento se toma en cuenta las siguientes consideraciones de acuerdo a la declaración de Helsinki así como el reglamento de la Ley general de Salud en Materia de Investigación para la Salud:

-Se conserva la intimidad y confidencialidad de la información personal de los sujetos que participan en la investigación. Esto se realizará al manejar todos los documentos sin incluir nombre de los sujetos ni datos personales. La información se recaba solamente con las iniciales del sujeto y el registro de su expediente. Toda información obtenida se resguardará en el Departamento de Infectología y solo tendrán acceso los investigadores, o las instancias que requieran hacer una auditoria del estudio, como lo son:

Comité de Investigación y Ética del HIMFG

Secretaria de Salud

-Si bien los datos consignados en el expediente se consideran datos personales sensibles sobre el estado de salud, está protegida por la ley de Transparencia y Acceso a la Información pública (Arts. 23,24 Fracción VI, 116 y 120) y la Ley Federal de Archivos (Arts. 26, 27 y 29). Así mismo en el artículo 120, Fracción V refiere que no se requerirá el consentimiento del titular de la información confidencial cuando se transmita la información entre sujetos obligados y entre estos y los sujetos de derecho internacional, en términos de los tratados y los acuerdos interinstitucionales, siempre y cuando la información se utilice para el ejercicio de facultades propias de los mismos. Por este motivo consideramos que no requiere de carta de consentimiento informado.

-Los documentos fuente se guardarán por al menos 10 años, conservando la privacidad de los mismos. La hoja de captura de datos se conservará por 5 años o hasta la publicación de los resultados de investigación.

## BIOSEGURIDAD

Consideramos que este estudio no tiene implicaciones de bioseguridad dado que se basa en la revisión de expedientes. Para fines del protocolo no se genera ningún tipo de residuos biológico-infeccioso ni CRETI. Todos los estudios de laboratorio que se tomaron o tomarán a los niños hospitalizados son de acuerdo con los estándares de atención clínica, los cuales no se modifican por este protocolo.

## REFERENCIAS.

1. Nicholson KG, Wood JM, Zambon M. Influenza. *Lancet*. 2003;362(9397). doi:10.1016/S0140-6736(03)14854-4.

2. Fisher, M C, J M, et al. *Sistemas de Vigilancia de Ifluenza Y Otros Virus Respiratorios En Las Américas: 2014.*; 2015.

3. Sistema Nacional de Información en Salud (SINAIS). Boletín de Información Estadística 2014 -2015. 2015:47.

4. Epidemiología DG de. INFORMACIÓN RELEVANTE DE INFLUENZA Información Semanal  Casos de IRA Casos de Neumonía / Bronconeumonía. 2017;2017.

BIBLIOGRAFÍA

1. Nicholson KG, Wood JM, Zambon M. Influenza. Lancet 2003;362:1733–45.
2. O’Brien MA, Uyeki T, Shay D, Thompson WW, Kleinman K, McAdam A, et al. Incidence of outpatient visits and hospitalizations related to influenza in infants and young children. Pediatrics 2004;113:585–93.
3. Neuzil KM, Mellen BG, Wright PF, Mitchel Jr EF, Griffin MR. The effect of influenza on hospitalizations, outpatient visits and courses of antibiotics in children. N Engl J Med 2000;342:225–31.
4. *Subsecretaria de Prevención y Promoción de la Salud. Centro Nacional para la Salud de la Infancia y la Adolescencia. Programa de Vacunación Universal y Semanas Nacionales de Salud. Lineamientos Generales 2017*.
5. Dirección general de Epidemiología. Anuarios de Mortalidad 2010-2016. <https://www.gob.mx/busqueda?utf8=%E2%9C%93&site=salud&q=anuarios+morbilidad> Last visit June 4th, 2017.
6. Hemming VG. Viral respiratory diseases in children: classification, etiology, epidemiology, and risk factors. J Pediatr 1994;124:S13–6.
7. Paisley JW, Lauer BA, McIntosh K, Glode MP, Schachter J, Rumack C. Pathogens associated with acute lower respiratory tract infection in young children. Pediatr Infect Dis 1984;3:14–9.
8. Henrickson KJ, Kuhn SM, Savatsky LL. Epidemiology and cost of infection with human parainfluenza virus types 1 and 2 in young children. Clin Infect Dis 1994;18:770–9.
9. WHO surveillance case definitions for ILI and SARI <http://www.who.int/influenza/surveillance_monitoring/ili_sari_surveillance_case_definition/en/> Last visit June 26^TH^ 2017.
10. Aranda-Romo S, Comas-García A, García-Sepúlveda CA, Hernández-Salinas AE, Piña-Ramírez M, Noyola DE. Effect of immunization program on seasonal influenza hospitalizations in Mexican children. Vaccine. 2010; 28 (13): 2550-2555
11. Lafond KE, Nair H, Rasooly MH, Valente F, Booy R, Rahman M, *et al*. (2016) Global Role and Burden of Influenza in Pediatric Respiratory Hospitalizations 1982-2012: A Systematic Analysis. PLoS Med 13(3):e1001977.
12. Silva ML, Perrier L, Späth HM, Grog I, Mosnier A, Havet N, Cohen JM and on behalf of the IBGP team. Economic burden of seasonal influenza B in France during winter 2010-2011. BMC Public Health 2014, 14:56
13. GBD 2015 Eastern Mediterranean Region LRI Collaborators & Mokdad, A.H. Int J Public Health. 2017. https://doi.org/10.1007/s00038-017-1007-0
14. de Francisco Shapovalova N, Donadel M, Jit M, Hutubessy R. A systematic review of the social and economic burden of influenza in low- and middle-income countries. Vaccine 2015; 33(48):6537–44.
15. WHO. WHO Manual for estimating the economic burden of seasonal influenza. Disponible en: http://www.who.int/immunization/research/development/influenza_maternal_immunization/en/index2.html
16. WHO. Metrics: Disability-Adjusted Life Year (DALY) Quantifying the Burden of Disease from mortality and Morbidity. Disponible en: http://www.who.int/healthinfo/global_burden_disease/metrics_daly/en/ consultado el 30 septiembre 2017.
17. World Health Organization 2004. The global burden of disease: 2004 update. Geneva: WHO.

Peasah SK, Azziz-Baumgartner E, Breese J, Meltzer MI, Widdowson MA. Influenza cost and costeffectiveness

studies globally – a review. Vaccine. 2013; 31(46):5339–48.

[9] Costa N, Derumeaux H, Rapp T, Garnault V, Ferlicoq L, Gillette S, et al. Methodological considerations

in cost of illness studies on Alzheimer disease. Health Econ Rev. 2012; 2(1):18.

[10] Ott JJ, Klein Breteler J, Tam JS, Hutubessy RC, Jit M, de Boer MR. Influenza vaccines in low and

middle income countries: a systematic review of economic evaluations. Hum Vaccin Immunother.

2013; 9(7):1500–11.

**Cuestionario sobre los costos asociados a la infección respiratoria aguda grave por influenza**

**Información del hospital**

1. Código del colector ________________

2. Nombre del hospital ________________

**Información del paciente**

3. ID de estudio del paciente ___________________

4. Nº de identificación del paciente. del expediente del hospital ________________

5. fecha de admisión _ _/ _ _/_ _ _ _

(Mes/ día/ año)

UCI o (Mes/ día/ año) Non-ICU

(Mes/ día/ año)

(Mes/ día/ año)

Fecha de descarga/muerte _ _/ _ _/_ _ _ _

(Mes/ día/ año)

7. fecha de nacimiento _ _/ _ _/_ _ _ _, Edad .

(Mes/ día/ año)

(Mes/ día/ año)

(Mes/ día/ año)

(Mes/ día/ año)

(Mes/ día/ año) (años de edad)

(Mes/ día/ año)

_ _Falta información

8. el paciente es de área:  urbano  rural  desconocido

9. Talla   .  cm

Peso   .  kg

10. Sexo: Hombre Mujer Falta

(Mes/ día/ año)

(Mes/ día/ año)

(Mes/ día/ año)

**Tratamiento paciente/historia diagnóstica**

11. Número de días de enfermedad antes de la hospitalización _____ Falta/desconocido

(Mes/ día/ año)

12. ¿Qué tipo de síntomas tuvo usted en ese momento en la enfermedad? (*Puede elegir más de una opción)*

Fiebre ≥ 38 ˚ C  Tos  Dolor de garganta  Se sintió febril (pero no sé ˚ C)

 Otros, especifique __________________________________________________________

_________________________________________________________________________

_________________________________________________________________________

_________________________________________________________________________

O  Falta/desconocido

Formulario de recolección de datos de muestra 1: formulario de utilización de atención médica hospitalaria

37

13. ¿recibió atención antes de llegar a esta instalación? (*Respuestas múltiples permitidas)*

 Falta/desconocido

 No

 Sí, por favor especifique dónde:

 Curandero tradicional  Herbolario

 Medicamentos de venta libre  Otra instalación similar a ésta

 Hospital de districto  Centro de salud

 Otros, especifique ____________________________________________________

Especifique los detalles de gastos para cada instalación seleccionada y el gasto total

_________________________________________________________________________

_________________________________________________________________________

_________________________________________________________________________

_________________________________________________________________________

_________________________________________________________________________

14. resultados sobre la descarga ___________________________________________________

 Vivo, bien  Vivo, parcialmente recuperado

 Vivo, pero desconocido del resultado/que falta  Se

 Dados de alta contra el Consejo médico  Fugado

 Murió  Falta/desconocido

15. ¿Cuál fue el diagnóstico de admisión? ________________________________________

16. ¿Cuál fue el diagnóstico final? _____________________________________________

17. ¿Cuántas co-morbilidades tuvo el paciente?  

*(esto incluye cualquier condición además de los diagnósticos primarios.)*

18. especificar las co-morbilidades ________________________________________________

_________________________________________________________________________

_________________________________________________________________________

Formulario de recolección de datos de muestra 1: formulario de utilización de atención médica hospitalaria

38

**Tratamiento del paciente/Diagnostic historia *(cont...)***

19. duración de la estancia por ubicación (duración récord de estancia en cada tipo de habitación, incluyendo

ceros). *Si la duración de la estancia es de 1 a 11 horas, escriba ½ Day. Si 12 – 24 horas, escriba 1 día.*

**Tipo de habitación duración de la estancia (días)**

 Clínica ambulatoria ________________________________

 Sala pediátrica ________________________________

 Unidad de cuidados intensivos o cuidados especiales ________________________________

 Unidad de aislamiento ________________________________

 Sala de emergencias ________________________________

 Otro (especifique) ________________________________

 Falta_

20. para esta visita, ¿cuántos parientes o cuidadores te acompañaron? _ _ persona (s)

(para pacientes hospitalizados, por favor especifique el número promedio de familiares o cuidadores que proveen atención

en un día)

21. ¿Qué pruebas diagnósticas se utilizaron? (*Marque las pruebas aplicables y anote el número de*

*pruebas realizadas. Si es posible, tenga en cuenta si cada prueba es para el diagnóstico o para el tratamiento)*

 **Ninguna prueba de diagnóstico**

 **Prueba de influenza para virus estacionales (no. de los tiempos de prueba realizados)**

 Prueba rápida, especifique los detalles de la prueba ________________________   tiempo (s)

 PCR, especifique los detalles de la prueba _____________________________   tiempo (s)

 Otros, especifique la prueba details____________________________   tiempo (s)

 **Conteo sanguíneo. (***Si el "sí", que uno (s)***)**

 Hematocrito/volumen lleno de la célula (HCT/PCV) _______________   tiempo (s)

 HB/hemoglobina ______________________________________   tiempo (s)

 Conteo sanguíneo completo, conteo sanguíneo completo, ____________________   tiempo (s)

 **Cultivo de sangre**

 **Prueba del VIH**

 Elisa_   tiempo (s)

 ___________________________________________ de prueba rápida   tiempo (s)

Formulario de recolección de datos de muestra 1: formulario de utilización de atención médica hospitalaria

**Tratamiento del paciente/Diagnostic historia *(cont...)***

21. ¿Qué pruebas diagnósticas se utilizaron? (*Marque las pruebas aplicables) (continuación)*

 **Radiología(***Si el "sí", que uno (s)***)**

 Tomografía computarizada (CT Scan) ____________________________   tiempo (s)

 Ultrasonido_   tiempo (s)

 Otros X-ray______________________________________________   tiempo (s)

 **Química sanguínea (***Si el "sí", que uno (s)***)**

 Electrolitos_   tiempo (s)

 Glucosa_   tiempo (s)

 **Otros (especifique el nombre de la prueba y el número de veces que se realiza la prueba):**

__________________________________________________________   tiempo (s)

__________________________________________________________   tiempo (s)

__________________________________________________________   tiempo (s)

__________________________________________________________   tiempo (s)

__________________________________________________________   tiempo (s)

__________________________________________________________   tiempo (s)

__________________________________________________________   tiempo (s)

__________________________________________________________   tiempo (s)
